# Searching for a Novel HLA-Cw6-Linked Cardiometabolic Endotype in Psoriatic Disease

**DOI:** 10.3390/biomedicines12102174

**Published:** 2024-09-25

**Authors:** Rubén Queiro, Pablo González del Pozo, Paula Alvarez, Norma Calleja, Ignacio Braña, Marta Loredo, Estefanía Pardo, Stefanie Burger, Sara Alonso, Mercedes Alperi

**Affiliations:** 1 Rheumatology Division, Central University Hospital of Asturias, 33011 Oviedo, Spain; pgd795@hotmail.com (P.G.d.P.); paulalvarez992@gmail.com (P.A.); normaale9222@gmail.com (N.C.); i.brana.abascal@hotmail.es (I.B.); mloredomart@gmail.com (M.L.); estefaniapardoc@gmail.com (E.P.); stefanie.nam@gmail.com (S.B.); saraalonsocastro@hotmail.com (S.A.); mercedes_alperi@hotmail.com (M.A.); 2 Department of Medicine, Oviedo University School of Medicine, 33006 Oviedo, Spain; 3 Translational Immunology Division, Biohealth Research Institute of the Principality of Asturias (ISPA), 33011 Oviedo, Spain; 4 Rheumatology & ISPA Translational Immunology Division, Hospital Universitario Central de Asturias, Avenida de Roma, S/N 33011, 33011 Oviedo, Spain

**Keywords:** psoriasis, psoriatic arthritis, psoriatic disease, HLA-Cw6, cardiometabolic comorbidity, diabetes

## Abstract

Background/Objectives: In recent years, a possible connection between HLA-Cw6 and a distinctive cardiometabolic (CM) profile in patients with psoriatic disease (PsD) has been proposed, although there is still little support for this. Our aim was to further investigate this possible association by studying a large population of PsD patients. Methods: For this study, three different cohorts of patients with PsD were analyzed: two with a majority of cutaneous psoriasis, pooled n: 600, and a third with only psoriatic arthritis—PsA—cases, n: 340. Potential relationships between HLA-Cw6 and the different CM risk factors (hypertension, diabetes, obesity, dyslipidemia) were analyzed using univariate and multivariate regression models, while the final net effect was assessed using fixed- or random-effects meta-analyses, as appropriate. Results: In the PsA cohort, no association was detected between HLA-Cw6 carriership and any of the CM comorbidity factors. In psoriasis cohorts, after correcting for age, sex, disease duration, and arthritis, HLA-Cw6 carriers had a reduced diabetes risk (OR 0.49, 95%CI: 0.26–0.91, *p* = 0.026). This latter effect was confirmed by a fixed-effects meta-analysis of the included cohorts (pooled OR: 0.50, 95%CI: 0.27–0.90). Conclusions: This work demonstrates a potential protective effect of the HLA-Cw6 allele on the risk of diabetes in PsD. Our findings together with those of others seem to confirm the existence of a novel HLA-Cw6-linked cardiometabolic endotype in this disease.

## 1. Introduction

Psoriasis and psoriatic arthritis (PsA) are the two main poles on which the modern concept of psoriatic disease (PsD) is based. However, this concept is also supported by the numerous comorbid conditions that accompany both entities, so today, it is understood that there is a common link between psoriasis, PsA, and their comorbidities [1]. Regarding the latter, the strongest pathogenic connections are, above all, with those of a cardiometabolic (CM) nature [2,3,4]. In fact, obesity is considered one of the long-term risk factors for the development of arthritis among patients with skin psoriasis [2,3,4]. Although it is speculated that the deregulation of the IL23-Th17 axis would be the basis for the coexistence of PsD and its CM comorbidity, it is no less true that a shared genetic basis could contribute to the appearance of skin and joint manifestations along with the CM conditions typical of PsD [5,6]. Therefore, the relationship between PsD and its CM comorbidity appears to be complex and possibly bidirectional, with chronic inflammation and immune dysregulation playing a critical role in the pathogenesis of both processes.

The histocompatibility antigen HLA-Cw6 is the main genetic biomarker of PsD. It not only establishes a clear risk of cutaneous psoriasis but is also associated with a specific disease endotype (early-onset, familial, severe skin disease). However, this allele is under-represented among arthritis patients, while it is also linked to a longer psoriasis arthritis latency time [7,8]. On the other hand, in the last few years, a potential association between HLA-Cw6 and a better CM risk profile has been established [9,10,11]. However, it is possible that these links are more related to confounders such as age, sex, and, above all, the presence of arthritis [12]. On the one hand, as mentioned, there is a negative relationship between HLA-Cw6 and arthritis. Furthermore, most CM comorbidity tends to be associated with increasing age. Finally, many studies associate PsA with a higher prevalence of both these factors and their core syndrome, the so-called metabolic syndrome [3,13,14]. Thus, the relationship between HLA-Cw6 positivity, skin involvement, early age of onset, and a better CM profile may actually be a mirrored relationship, where the other side of the mirror would be a negative HLA-Cw6 profile, later age of onset, worse CM profile, and arthritis. Thus, the question remains as to whether these potential connections between HLA-Cw6 and a lower risk of CM comorbidity are rather indirect relationships, where other factors such as age or arthritis better explain this entire apparent connection.

In previous studies, we have found an association between HLA-Cw6 carriership and a better CM risk profile, but we have also found that other factors (e.g., age, sex, arthritis) could better explain this connection [10,12]. However, in these and other studies, the majority of the study population has been patients with psoriasis without arthritis, so we do not know whether enriching these analyses by including more patients with PsA could contribute to shedding light on this controversial aspect of the disease. In the present study, which includes a large number of patients, we delve deeper into the possible connections between HLA-Cw6 and CM risk factors but in a more balanced population in terms of the representativeness of PsA cases.

## 2. Materials and Methods

### 2.1. Study Population

For the purposes of this study, we analyzed two cohorts of patients mostly represented by cutaneous psoriasis cases and a third cohort, strictly represented by patients with PsA according to CASPAR criteria [15]. All patients included were over 18 years of age and were originally from a region in northwestern Spain (Principality of Asturias, total population of around one million inhabitants). Patients were recruited from the Dermatology and Rheumatology services of two university hospitals in that region. The recruitment period for these cohorts ran from January 2007 to December 2017. The ethical considerations, inclusion/exclusion criteria, main characteristics, and scientific exploits, relevant to these cohorts, were published elsewhere [10,12,16]. Also, aligned with the study objectives, the first psoriasis cohort consisting of 400 patients [10] was merged with the second cohort consisting of 572 cases [12], but after checking for replicate data, the final psoriasis study cohort consisted of 600 patients, keeping the third cohort of patients with PsA (n: 340) separate. Approximately 30% of patients included in the psoriasis cohort met CASPAR criteria for PsA, although this subpopulation was not characterized in detail, and its presence was simply recorded (dichotomous variable). This study was conducted in full conformance with the Spanish SAS Order/3470/2009 of the Ministry of Health and Social Policy, local laws and regulations, and the ethical principles laid down in the Declaration of Helsinki. Compliance with the provisions of the new Regulation (EU) 2016/679 of the European Parliament and the Council of 27 April 2016 on Data Protection (GRDP) was also ensured.

### 2.2. Study Variables

Sociodemographic and anthropometric data and laboratory, activity, and outcome variables were included. The details of these variables and their definitions were published elsewhere [10,12,16]. Briefly, a family history of psoriasis and arthritis, age, sex, educational level, anthropometric data, disease duration, psoriasis types, nail involvement, BSA, PASI, arthritis patterns, associated manifestations (dactylitis, enthesitis, uveitis, IBD), composite arthritis activity indices, physical function, and data on structural damage were collected. Cardiometabolic comorbidities collected were obesity, hypertension, diabetes, and dyslipidemia. Accepted definitions for these comorbidities were previously published [16].

### 2.3. HLA-Cw6 Typing

All patients were genotyped for the HLA-Cw6*0602 allele (SNP rs1050414 C/G).

### 2.4. Statistical Analysis

For the univariate descriptive analysis, the mean, standard deviation, median, minimum and maximum value of the quantitative variables, and the absolute and relative frequencies (percentages) of the categorical variables were included. For comparisons between groups and variables, parametric and non-parametric tests were chosen depending on whether their distribution was normal or not. In the PsA cohort, the frequency of CM factors was compared to that of 600 outpatients with non-inflammatory characteristics matched by age (± 3 years) and sex (1:1) with the study population. To estimate the crude effect of the genetic marker (HLA-Cw6) on the different outcomes, simple linear regression models were constructed when the dependent variable was a quantitative one, and simple logistic regression models were constructed when the dependent variable was categorical. To estimate the adjusted effect, multiple regression models were created (linear or logistic, as appropriate) where covariates such as age, sex, time of evolution, and the presence of arthritis were introduced. Finally, to assess the net effect of the relationship between HLA-Cw6 and the different CM factors, meta-analyses were carried out, presenting the pooled Odds Ratio (OR) for fixed- and random-effect models. In the fixed-effects model, it is assumed that the study populations were the same. This model can be used when there is no heterogeneity between studies (*p* for heterogeneity > 0.05). The threshold for statistical significance was set at *p* < 0.05. Data were analyzed using R software (4.3.1 “Beagle Scouts”).

## 3. Results

### 3.1. A Summary of the Study Population

The total population included 940 patients, 600 from the two merged psoriasis cohorts and 340 from the arthritis cohort. The psoriasis study cohort included 312 (52%) men and 288 (48%) women, mean age 46.7 ± 14.5 years, average disease duration of 20 ± 14.8 years. Psoriatic arthritis cohort included 190 (55.9%) men and 150 (44.1%) women, mean age 55 ± 13 years, average joint disease duration of 11 ± 6.3 years. The rest of the characteristics of the pooled psoriasis cohorts and the PsA cohort are summarized in Table 1.

### 3.2. Cardiometabolic Comorbidity in the PsA Cohort

The frequency of traditional CM factors was as follows: diabetes 13.8%, hypertension 36%, dyslipidemia 31%, obesity 35%, and overweight 24.1%. Compared to the control non-PsA population, PsA patients showed a higher frequency of CM comorbidity: hypertension (36 vs. 23%, OR 2.4, 95% CI: 1.6–2.7, *p* < 0.0001), diabetes (13.8 vs. 5%, OR 2.8, 95% CI: 1.7–4.3, *p* < 0.0001), obesity (35 vs. 22%, OR 2.1, 95% CI: 1.5–2.8, *p* < 0.0001), and tobacco use (26 vs. 21%, OR 1.4, 95% CI: 1.0–1.8, *p* < 0.05). More females (18%) than males had diabetes (10.5%), *p* < 0.05. Also, more females (44%) than males (28.9%) were obese, *p* = 0.01.

### 3.3. Cardiometabolic Comorbidity in Psoriasis Cohorts

The average BMI was 27.7 (SD 5.04), while the average abdominal perimeter was 97 cm (min: 60, max: 138). The classic cardiovascular risk factors showed the following prevalences: smoking (34%), non-alcoholic fatty liver disease—NAFLD—(22%), hypertension (20%), dyslipidemia (20%), and diabetes (11.4%). In total, 33 patients showed adverse coronary events (5.5%). More men (32%) than women (11.4%) had NAFLD (*p* < 0.01), while there were no significant differences in the distribution of the other CM risk factors.

### 3.4. Relationship between HLA-Cw6 and Cardiometabolic Comorbidity

The determinants of diabetes, obesity, hypertension, and dyslipidemia in the PsA cohort have been published elsewhere [16]. However, all of these factors also appeared to be linked to increasing age: hypertension OR 1.19 (95%CI: 1.09–1.30, *p* < 0.001), diabetes OR 1.12 (95%CI: 1.04–1.23, *p* = 0.006), dyslipidemia OR 1.08 (95%CI: 1.03–1.14, *p* = 0.002), and obesity OR 1.07 (95%CI: 1.01–1.15, *p* = 0.02). On the other hand, no association was detected between the HLA-Cw6 allele and any CM factor. Increasing age was also a driving force to explain CM comorbidity in psoriasis cohorts, but it was not the only one. Although age increased the risk of hypertension (OR 1.11; 95% CI: 1.08–1.13; *p* < 0.001), arthritis also independently increased this risk (OR 1.59; 95% CI: 1.02–2.61; *p* < 0.05). However, no link was found between this risk factor and HLA-Cw6 positivity after adjusting for age, sex, disease duration, and arthritis (OR 0.79, 95%CI: 0.47–1.30, *p* = 0.4). Diabetes risk increased with age (OR 1.08, 95%CI: 1.05–1.10, *p* < 0.001), but HLA-Cw6 carriers also reduced that risk after multiple testing (OR 0.49, 95%CI: 0.26–0.91, *p* = 0.026). The two factors independently associated with dyslipidemia in multivariate regression models were age (OR 1.06, 95%CI: 1.05–1.08, *p* < 0.001) and arthritis (OR 1.82, 95%CI: 1.15–2.87, *p* = 0.010). The risk of NAFLD increased by 2% for each decade of age (*p* = 0.02) and was reduced by 74% in women compared to men (*p* < 0.001). Adverse coronary events increased by 11% per decade (*p* < 0.001).

### 3.5. Meta-Analysis of Relationships between HLA-Cw6 and Cardiometabolic Comorbidity

**Figure 1:**
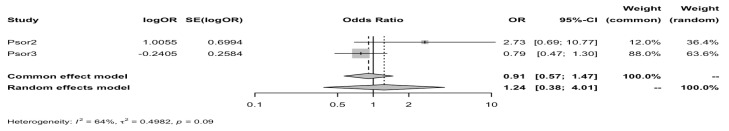
A forest plot of the relationship between HLA-Cw6 and hypertension risk**.** Pooled ORs are presented for both the fixed-effects and random-effects models. Psor2: psoriatic arthritis cohort. Psor3: pooled psoriasis cohorts. See text for details.

No heterogeneity was detected between the cohorts (*p* for heterogeneity > 0.05), so a fixed-effects meta-analysis model was applied. In the case of hypertension, some heterogeneity was detected between cohorts (*I*^2^ 64%), but the precept to use a fixed-effects meta-analysis was fulfilled (*p* for heterogeneity = 0.09). As shown in Figure 1, no relationship was found between HLA-Cw6 and the risk of hypertension (pooled OR 0.91, 95%CI: 0.57–1.47).

**Figure 2:**
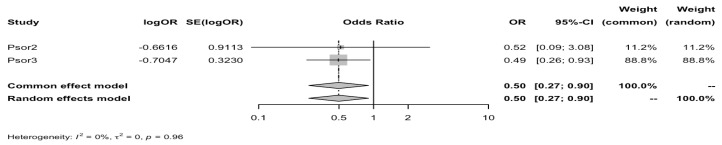
A forest plot of the relationship between HLA-Cw6 and diabetes risk**.** Pooled ORs are presented for both the fixed-effects and random-effects models. Psor2: psoriatic arthritis cohort. Psor3: pooled psoriasis cohorts. See text for details.

A significant inverse association was found between HLA-Cw6 and diabetes with a pooled OR 0.50, 95%CI: 0.27–0.90 (Figure 2).

**Figure 3:**
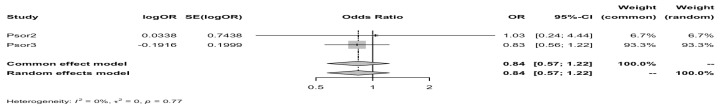
A forest plot of the relationship between HLA-Cw6 and obesity. Pooled ORs are presented for both the fixed-effects and random-effects models. Psor2: psoriatic arthritis cohort. Psor3: pooled psoriasis cohorts. See text for details.

As shown in Figure 3, a 16% reduction in obesity risk was found among HLA-Cw6 carriers, but this was not significant (95%CI: 0.57–1.22).

**Figure 4:**
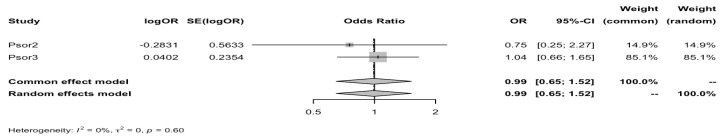
Pooled ORs are presented for both the fixed-effects and random-effects models. Psor2: psoriatic arthritis cohort. Psor3: pooled psoriasis cohorts. See text for details.

Finally, as shown in Figure 4, no relationship was found between HLA-Cw6 and dyslipidemia (pooled OR 0.99, 95%CI: 0.65–1.52).

## 4. Discussion

In this study, which included a large number of patients with PsD, we detected an inverse association between HLA-Cw6 and diabetes (OR 0.50). Although this association was primarily detected in cohorts of patients with predominantly skin psoriasis, a fixed-effect meta-analysis including all cohorts confirmed the lower risk of diabetes associated with HLA-Cw6 carriership. It is also interesting to note that although HLA-Cw6 was over-represented in women with cutaneous psoriasis (46% vs. 39%), sex did not play a relevant role in the associations found in this study.

Some studies conducted in the last few years have detected an inverse association between the HLA-Cw6 allele and certain cardiovascular risk factors. Thus, at least two independent studies have linked the presence of this marker with a lower risk of hypertension [9,10]. This allele has also been linked to lower central adiposity and a lower risk of liver fibrosis, thus driving a potential protection conferred by HLA-Cw6 against a higher risk of cardiovascular comorbidity [9,10,11,17]. However, none of these studies have been able to definitively resolve the possibility of a false causal relationship between this marker and this type of comorbidity. Among other factors, the populations analyzed have been very different and mostly made up of patients with psoriasis without arthritis, the prevalence of HLA-Cw6 has also been quite heterogeneous, and the prevalence of the disease itself and its comorbidities has been uneven between studies, so comparisons between these studies are quite complex. We even found a certain disparity in our own previous results, such that the associations in this regard found in the first study were not confirmed in a second larger study [10,12]. Therefore, the central idea of the present work was to seek a certain breaker in relation to our previous findings. To this end, we meta-analyzed three cohorts of patients (two merged into one, with a majority representation of psoriasis without arthritis, and a third, composed exclusively of patients with PsA). After correcting for age, sex, disease duration, and arthritis, we confirmed a “protective” effect against diabetes conferred by HLA-Cw6.

Both PsD and diabetes are polygenic diseases resulting from a complex interplay between genetic, epigenetic, and environmental factors. In the case of diabetes, most experts agree that the HLA system is the principal region of risk for developing type 1 diabetes, and it is less important for type 2 [18,19]. Experimental research shows evidence of the biochemical consequences of the nonenzymatic reaction of oxidative alterations in key components of the Major Histocompatibility Complex (MHC) in vivo under conditions of hyperglycemia-induced metabolic stress. These modifications were linked to epitope-specific changes in endosomal processing efficiency, MHC-II peptide binding, and editing activity [20]. These findings highlight a potential link between glycation reactions and altered MHC antigen presentation that may contribute to type 2 diabetes complications. Under these premises, it would be tempting to speculate on a possible pathogenic link between HLA-Cw6 and the risk of diabetes in the PsD population. Indeed, a recent study by Mendoza-Ramírez et al. found links between certain HLA-C alleles (C*01:02:01:01) and the risk of type 2 diabetes, but HLA-C*06 (HLA-Cw6) was not among the risk alleles [21]. It would therefore be interesting to delve deeper into the mechanisms of this protective relationship, perhaps by analyzing other omics profiles in serum or urine. Therefore, until we have these kinds of studies, the biological plausibility of our finding will remain uncertain.

On the other side, there are some epidemiological clues that might support this connection. Eirís et al. demonstrated that genetic variations in the IL12/23 pathway were important not only for defining both the risk and severity of PsD but also for defining the risk of certain comorbidities such as type 2 diabetes. Thus, this group found significant associations between three SNP genotypes and type 2 diabetes, IL12B rs6887695-CC (OR = 2.90), IL12B rs3212227-CC (OR = 5.90), and IL23R rs2201841-GG (OR = 2.69), demonstrating this way that the genetic risk of PsD-related diabetes might be governed by genes outside the HLA system [22]. Inerot et al. found endocrine disorders in 9% of their HLA-Cw6-negative psoriasis patients compared to only 1% among HLA-Cw6 carriers. In addition, no HLA-Cw6-positive patient had diabetes compared to the 5% overall prevalence of diabetes in the Swedish general population [23]. The latter seems to reinforce our findings on the protective potential of HLA-Cw6 in the risk of PsD-related diabetes. In Douroudis et al.’s study, the five CM comorbidities studied (ischemic heart disease; hypertension; dyslipidemia; type 2 diabetes; and a cardiovascular disease umbrella, including ischemic, cerebrovascular, and peripheral vascular disease) all exhibited lower prevalence in HLA-Cw6-positive patients with psoriasis than in HLA-Cw6-negative patients. However, only hypertension remained statistically significant after accounting for multiple testing (OR 0.73 in HLA-Cw6 carriers) [9]. These latest findings, together with our own, do seem to point to a relationship between HLA-Cw6 carriership and a better cardiovascular risk profile. Furthermore, despite the complex interaction between the disease and its cardiovascular comorbidities, our results may send a message for practice, as this specific type of comorbidity is increasingly helping physicians to better plan therapy for patients with PsD [24,25].

The final consideration regarding the protective role of HLA-Cw6 against CM comorbidity is whether this could translate into a lower rate of future adverse cardiovascular events among HLA-Cw6 carriers. As this is an open question, it is worth highlighting that in a study by Eder et al., interestingly, HLA-B*13:02 and HLA-C*06:02 (HLA-Cw6) were associated with more severe atherosclerosis (age- and sex-adjusted OR 2.31 and OR 1.68, respectively), and these associations remained statistically significant after adjusting for cardiovascular risk factors [26].

In short, the HLA-Cw6-linked endotype has been gradually expanding, moving from a classic clinical endotype (type I disease), linked to a higher family burden, early onset, and greater clinical severity, to a pharmacogenomic endotype, linked to a better response to certain therapies (methotrexate and ustekinumab), to finally arrive at a novel cardiometabolic endotype that, if confirmed in other latitudes, would have clear implications for the overall management and prognosis of the disease. Therefore, it may be time to incorporate HLA-Cw6 determination into clinical routine in order to improve the standard of care in this field.

This study obviously has several limitations. First, since the different outcomes analyzed and their results are subject to multiple influences, it may be the case that multivariate models cannot fully account for the influence of all possible mediating variables in all cases. Furthermore, the cross-sectional nature of the observations limits our ability to draw causal inferences. Moreover, the use of a meta-analysis to obtain the net effect of the investigated associations is not without criticism in this particular context, including the potential bias inherent in the analysis of cohorts from the same working group thus limiting its quality. An additional weakness is that each cardiometabolic factor was analyzed separately, when it is well known that in clinical reality, many of these factors coexist in the same patient (metabolic syndrome). That is, an interaction analysis was not provided in this regard. Given these uncertainties, the associations found in our work should be confirmed in future adequately powered life course studies. As advantages, we included a significant number of cases with an adequate representation of one of the usual confounding factors in this type of association studies, such as arthritis.

Finally, it must be remembered that the relationship between PsD, its comorbidities, and their genetic drivers cannot be seen as a unidirectional relationship but rather as a result of multiple interactions [27]. A good example of this can be found in the relationship between tobacco and psoriasis, where in addition to promoting oxidative damage and expanding cell lines involved in pathogenesis, tobacco may enhance the expression of genes involved in the etiology of the disease, such as HLA-Cw6 [28].

## 5. Conclusions

In summary, our work demonstrates a potential protective effect of the HLA-Cw6 allele on the risk of diabetes that was independent of the presence of arthritis and other confounders. Since the distribution of HLA-Cw6 is not homogeneous worldwide, additional well-designed studies from other latitudes are needed to support the existence of this novel HLA-Cw6-linked cardiometabolic endotype. It remains to be seen whether the benefits of this positive cardiometabolic endotype would be associated in the future with better cardiovascular outcomes in this entity.

## Figures and Tables

**Table 1:** Disease characteristics of study cohorts**.**

Variables	PsA. N: 340	Psoriasis. N: 600 *
Age (yr ± SD)	55 ± 13	46.7 ± 14.5
Age at onset (yr ± SD)		
Skin disease	33 ± 17	23 ± 12.4
Joint disease	45 ± 14	
Disease duration:		
Skin disease (yr ± SD)	21 ± 11	20 ± 14.8
Joint disease (yr ± SD)	11 ± 6.3	
Men (n, %)	190 (55.9)	312 (52)
Women (n, %)	150 (44.1)	288 (48)
Education level:		
Primary (n, %)	180 (52.9)	168 (28)
Secondary (n, %)	85 (25)	276 (46)
Academic (n, %)	75 (22.1)	156 (26)
Psoriasis features:		
Plaque psoriasis (n, %)	295 (86.7)	522 (87)
Onychopathy (n, %)	142 (41.8)	342 (57)
Psoriasis in ≥3 body areas (n, %)	150 (44.1)	PASI ≥ 10: 306 (51)
Family history:		
Psoriasis (n, %)	156 (46)	242 (40.3)
PsA (n, %)	51 (15)	50 (8.3)
Joint pattern:		
Mono/oligoarthritis (n, %)	141 (41.5)	
Polyarthritis (n, %)	95 (28)	
Axial disease (n, %)	20 (5.9)	
Mixed (n, %)	80 (23.5)	
PsA features:		
Dactylitis (n, %)	93 (27.4)	
DIP joint disease (n, %)	74 (21.8)	
Mutilating arthritis (n, %)	5 (1.5)	
Erosive disease (n, %)	68 (20)	
HLA-Cw6 (n, %)	129 (38)	255 (42.5)
Treatment:		
Conventional systemics (n, %)	221 (65)	370 (61.7)
Biologics (n, %)	146 (43)	240 (40)

PsA: psoriatic arthritis, Yr: years, SD: standard deviation, N/n: numbers, PASI: psoriasis area and severity index, DIP: distal interphalangeal, HLA: human leukocyte antigen. * Upon multiple logistic regression analyses for the presence of PsA, significant associations were found with psoriasis severity (OR: 2.14, 95%CI: 1.46–3.16), female sex (OR: 1.63, 95%CI: 1.12–2.38), and the IFIH1/MDA5 rs1990760 TT genotype (OR: 1.62, 95%CI: 1.11–2.37), while HLA-Cw6 carriership was protective (OR: 0.65, 95%CI: 0.44–0.95) [12].

## Data Availability

The materials and raw data described in this manuscript will be made freely available to any researcher without breaching any participant’s confidentiality. To facilitate the revision of the results by other researchers, a file with the patient data is available as an Excel file upon request to the corresponding author.
